# Downregulation of Polyamine and Diamine Oxidases in Silicon-Treated Cucumber

**DOI:** 10.3390/plants10061248

**Published:** 2021-06-19

**Authors:** Anita Szegő, Iman Mirmazloum, Zsolt Pónya, Oyuntogtokh Bat-Erdene, Mohammad Omran, Erzsébet Kiss-Bába, Márta Gyöngyik, István Papp

**Affiliations:** 1 Department of Plant Physiology and Plant Ecology, Institute of Agronomy, Hungarian University of Agriculture and Life Sciences, Ménesi Str. 44, 1118 Budapest, Hungary; Szego.Anita@uni-mate.hu (A.S.); oyuntogtokhb@gmail.com (O.B.-E.); mohammad.omran1994@gmail.com (M.O.); Kissne.Baba.Erzsebet.Ilona@uni-mate.hu (E.K.-B.); Gyongyik.marta@gmail.com (M.G.); papp.istvan@uni-mate.hu (I.P.); 2 Division of Applied Food Crop Production, Department of Agronomy, Institute of Agronomy, Kaposvár Campus, Hungarian University of Agricultural and Life Sciences, Guba Sándor Str. 40, 7400 Kaposvár, Hungary; ponyazs@yahoo.com

**Keywords:** polyamine oxidase, diamine oxidase, reactive oxygen species, silicate, *Cucumis sativus*

## Abstract

Silicon (Si) is a ubiquitous element in soil with well-known beneficial effects under certain conditions, in several plant species, if supplied in available form for uptake. It may alleviate damage in various stress situations and may also promote growth when no obvious stressors are applied. Effects of Si are often linked to mitigation of oxidative stress, in particular to the induction of antioxidant defense mechanisms. In the work presented, the impact of silicon provision on pro-oxidant systems was investigated in cucumber. Plants of the F1 cultivar hybrid ‘Joker’ were grown under in vitro conditions in the absence of any applied external stressor. Silicon provision decreased H_2_O_2_ content and lowered lipid peroxidation in the leaves of the treated plants. This was paralleled by declining polyamine oxidase (PAO) and diamine oxidase (DAO) activities. Several PAO as well as lipoxygenase (LOX) genes were coordinately downregulated in Si-treated plants. Unlike in similar systems studied earlier, the Si effect was not associated with an increased transcript level of gene coding for antioxidant enzymes. These results suggest an inhibitory effect of Si provision on pro-oxidant amine oxidases, which may decrease the level of reactive oxygen species by retarding their production. This extends the molecular mechanisms linked to silicon effects onto redox balance in plants.

## 1. Introduction

Silicon (Si) is an abundant element in the earth’s crust, which may be necessary and can have beneficial effects in several plant species in varying degrees [1,2]. Grasses typically display high silicon level, while cucumber is a moderate Si accumulator [3]. Si may alleviate damage under stress conditions, but it can also improve growth in apparently stress-free situations [4,5]. In plants, silicon interacts with various cell wall components through biosilicification [6]. This results in direct effects such as mechanical strengthening, but indirect physiological consequences are also becoming evident. In biotic interactions, for example, Si nutrition triggered the elevated resistance of miniature roses towards the fungus *Podosphaera pannosa*. This effect was attributed to the accumulation of antifungal phenolic compounds [7]. As a concept for providing a general explanation of the diverse physiological effects of silicon, the ‘apoplastic obstruction hypothesis’ has recently been put forward [8]. According to this model, Si may serve as an extracellular ‘prophylactic agent’, inhibiting cell wall related reactions/interactions. Observed effects of Si provision may ultimately be due to these apoplastic interferences with diverse downstream consequences through cascading molecular events. Beneficial effects of silicon treatment have frequently been studied in relation to abiotic stresses. In a well-characterized example, the salt stress tolerance of cucumber was improved by Si treatment, which was ascribed to increased antioxidant enzyme activities and polyamine accumulation [9,10]. Polyamine metabolism is active in the extracellular space; in particular, amine oxidases contribute to production of reactive oxygen species in this compartment [11,12]. Besides a traditional role in wound response, lipoxygenases may also contribute to shaping the redox balance in diverse abiotic stress conditions [13]. In the work presented here, we set out to investigate whether the regulation of amine oxidases and lipoxygenases may display any characteristic patterns corresponding to redox balance in response to Si treatment in cucumber.

## 2. Results

### 2.1. Plant Growth and Silicate Content

**Figure 1:**
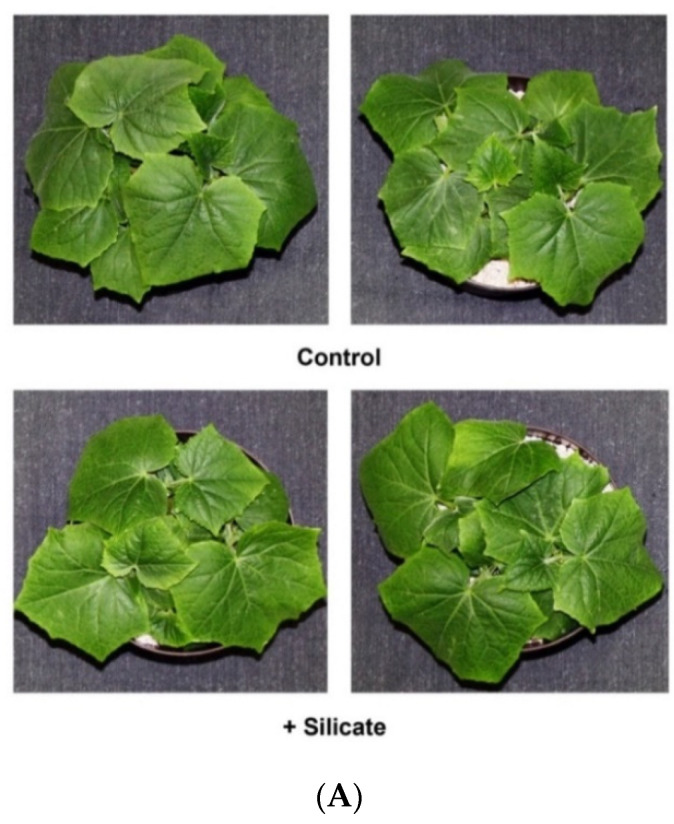

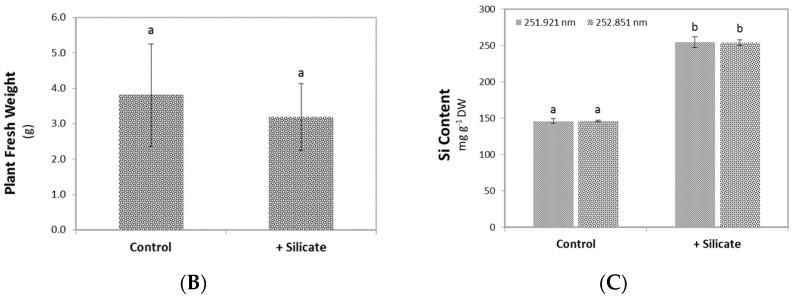
(**A**): ‘Joker’ plants grown in vitro without (upper panel) and with (lower panel) silicate-supplemented fertigation; (**B**): Fresh weight; and (**C**): silicon content of the plants at the end of the experiment. Values are means ± SD and different letters above the bars indicate significant difference between the treatments (Tukey post hoc: *p* < 0.05); *n* = 20.

Silicate treated plants did not display any major phenotypic alterations when compared to controls during the experimental period (Figure 1A). Growth of treated plants was not significantly different by the end of the experiment (Figure 1B). The Si concentration increased significantly in treated plants. Leaves of plants with Si-supplemented fertigation accumulated 254.69 mg.g^−1^ DW of silicon, whereas the concentration of Si in control plants was 146.05 mg.g^−1^ DW (Figure 1C).

### 2.2. Bio-Photon Emission Imaging

**Figure 2:**
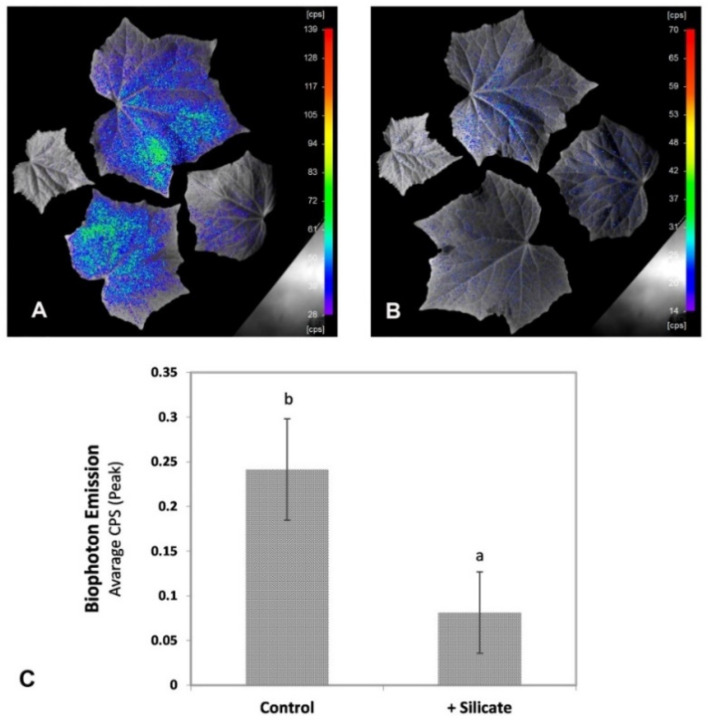
False colored images based on bioluminescence of typical (**A**): control and (**B**): silicate-treated plants. (**C**): Results of bio-photon emission (average count per second) in control and silicate-treated cucumber leaves. Bio-photon emission decreased significantly in response to Si treatment. Values are means ± SD and different letters above the bars indicate significant difference between the treatments (Tukey post hoc: *p* < 0.05); *n* ≥ 50.

Plants displayed moderate levels of spontaneous bio-photon emission under the growth conditions applied, in the absence of any particular stress treatment. Quantification approved substantially and significantly (*p* < 0.05) lower levels of bio-photon emission from the leaves of plants treated with silicon (Figure 2).

### 2.3. H_2_O_2_ Content and Lipid Peroxidation

**Figure 3:**
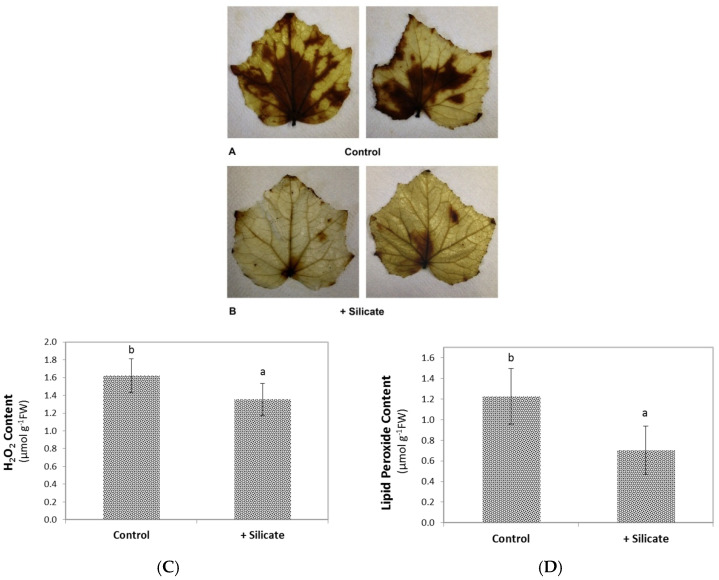
DAB staining of cucumber leaves of (**A**): control and (**B**): silicate-treated plants. (**C**): H_2_O_2_ and (**D**): lipid peroxide content of control and Si-treated plants. Values are means ± SD and different letters above the bars indicate significant difference between the treatments (Tukey post hoc: *p* < 0.05); *n* = 8.

Control plants displayed moderate coloration by DAB staining under the growth conditions applied. This staining disappeared along with a significant decline (*p* < 0.05) of H_2_O_2_ content (from 1.624 µM to 1.353 µM per gram fresh weight) when cucumber plants were treated with silicon (Figure 3A–C). The lipid peroxide content of the leaves also decreased significantly (*p* < 0.05), from 1.227 to 0.704 µM.g^−1^ FW, due to the Si treatment (Figure 3D).

### 2.4. Polyamine Oxidase, Diamine Oxidase, and Ascorbate Peroxidase Enzyme Activities

**Figure 4:**
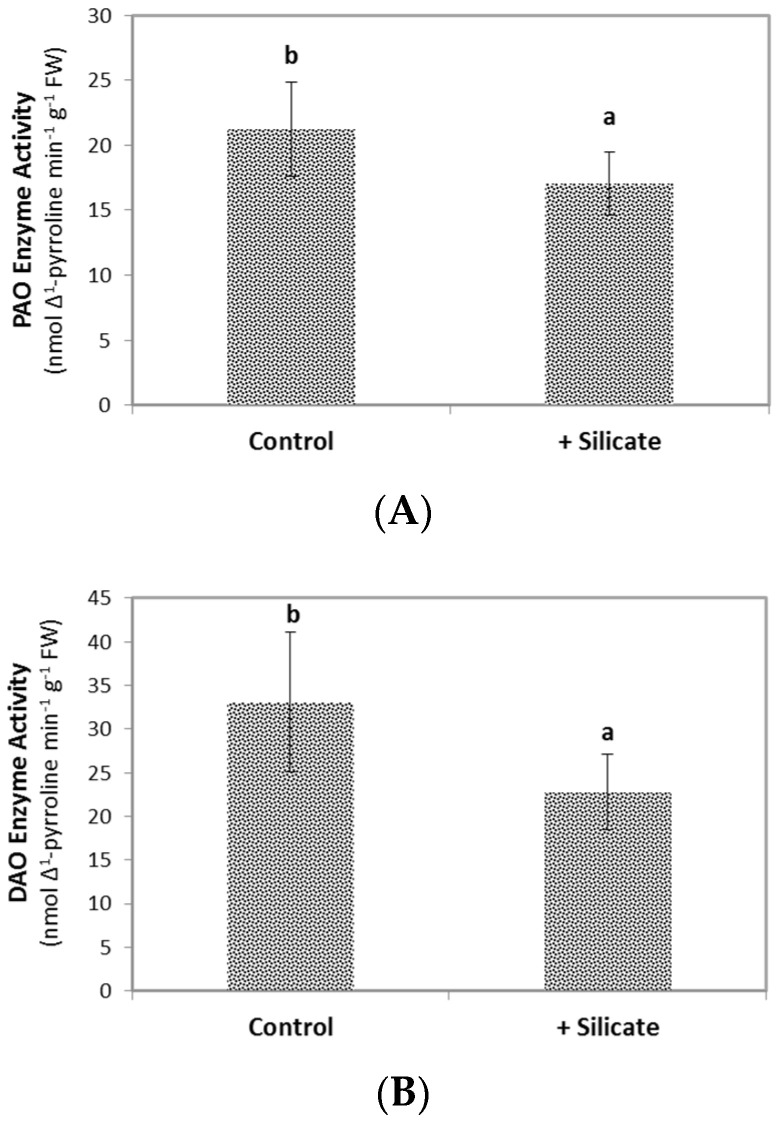

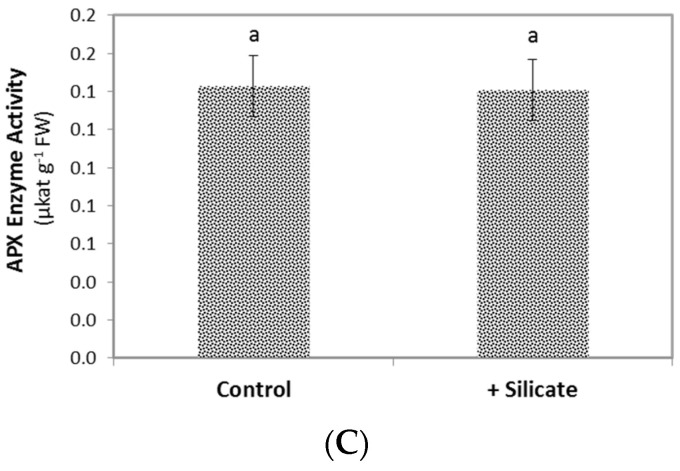
(**A**): PAO (Polyamine Oxidase); (**B**): DAO (Diamine Oxidase); and (**C**): APX (Ascorbate Peroxidase) enzyme activities in leaves of control and Si-treated cucumber plants. Values are means ± SD and different letters above the bars indicate significant difference between the treatments (Tukey post hoc: *p* < 0.05); *n* = 8.

As presented in Figure 4A,B, the polyamine oxidase (PAO) and diamine oxidase (DAO) enzyme activities in cucumber leaves declined significantly (*p* < 0.05) as a result of Si-supplemented fertigation, while the ascorbate peroxidase (APX) activity remained unchanged (Figure 4C).

### 2.5. Transcriptional Analysis of Selected Redox-Related Genes

Based on published data and the cucumber genomic sequence, several selected genes have been implicated or presumed to function in redox protection in cucumber. The ascorbate peroxidase genes (*APX1* and *APX2*) were taken from the cucurbit genomic database (http://cucurbitgenomics.org/, accessed on 1 October 2020) by keyword search for coding mRNA sequences and were selected based on the gene expression data of deposited bio projects. The glutathione peroxidase (*GPX1*) was selected from the work of Zhou et al. [14] due to its exceptionally high responsiveness and its expression recorded on NaCl and PEG treatment in their study. Two glutathione reductase (*GR1* and *GR2*) and four polyamine oxidase genes were found for cucumber after mining the cucurbit genome data base. The lipoxygenase genes were selected based on their reported expression in cucumber leaves by Yang et al. [15]. 

The expression of the selected *APX*, *GPX*, *GR*, *PAO,* and *LOX* genes was tested by semi-quantitative RT-PCRs. All reactions were performed on the same amount of cDNA, and yielded products of the expected fragment sizes (Table S1). 

**Figure 5:**
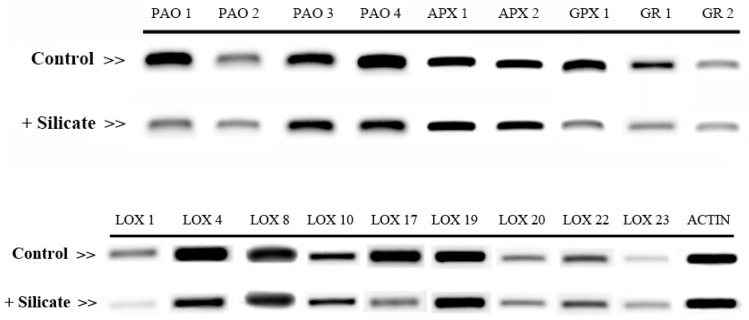
Semi quantitative RT-PCR results of selected genes affected by Si treatment from cucumber leaf samples in comparison with control plants.

According to the semi-quantitative RT-PCR data presented in Figure 5, all selected genes were found to be downregulated in response to silicate treatment as compared to the control actin gene (Figure 5).

In order to investigate the regulation of pro-oxidant systems, gene coding for PAO and LOX enzymes were selected as described above and their expression level was studied upon Si supplementation fertigation. Despite vigorous database searches and evidence for DAO activity in cucumber (see above), we could not identify putative *DAO* genes in the cucumber genome.

**Figure 6:**
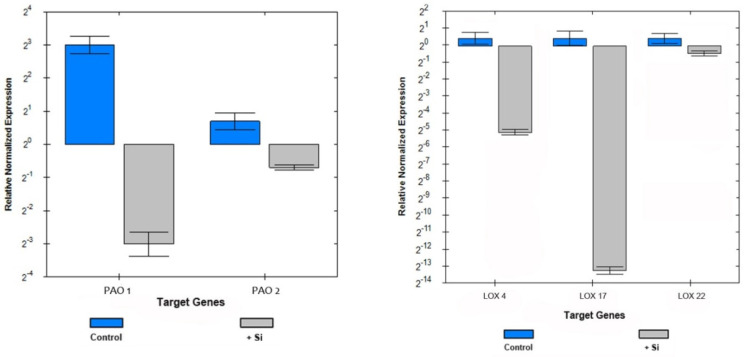
RT-qPCR data for transcript abundance of the *PAO1*, *2* and *LOX 4*, *17*, *22* genes in control and silicon-treated leaf samples.

In order to validate the semi-quantitative RT-PCR results, several representative genes were subjected to RT-qPCR analysis. RT-qPCR data for the *PAO 1*, *2* and *LOX 4*, *7*, *22* genes fully confirmed the results obtained by the RT-PCR method (Figure 6).

## 3. Discussion

Silicon (Si) is an element with potential benefits and variable accumulation rate in different plant species [3,16]. Cucumber is a moderate accumulator of Si, which has already been used as a dicot model system for Si effects [17]. A well-known aspect of alleviating environmental stresses by Si is the decrease in ROS level, which has been attributed so far to the increased expression and activity of antioxidant enzymes. This has been shown in various experimental systems, including salt-stressed cucumber [4,5,9]. In an attempt to further elaborate mechanisms of silicate action, we aimed to elucidate whether pro-oxidative systems, especially amine oxidases and lipoxygenases, might be affected by Si exposure.

In order to keep potentially interfering physiological processes at the minimum, we did not apply stress treatments to the experimental plants. Instead, we studied non-stressed plants of the F1 cultivar hybrid ‘Joker’, which displayed low-level hydrogen peroxide accumulation and lipid peroxidation in the leaves under the applied in vitro growth conditions. H_2_O_2_ content was measured directly, while lipid peroxidation was assessed by determining specific metabolites and also by bio-photon imaging, which is a powerful, non-destructive method serving this purpose [18]. Upon silicon provision, bio-photon imaging, H_2_O_2_ content, and direct measurement of lipid peroxidation demonstrated that Si treatment alleviated oxidative stress (Figure 3, Figure 4 and Figure 5 respectively). For an explanation of the mild oxidative stress observed in the plants, we note that ‘Joker’ is a hybrid bred for and usually grown in open field cultivation. Its root system is adapted for growth in open soil rather than in the confined space available in the pots. This situation probably represents physical stress [19], potentially provoking a systemic response [20] and may therefore be responsible for the mild oxidative burden in shoots of otherwise non-stressed ‘Joker’ plants. Seedlings may have also experienced slightly hypoxic conditions when growing in Rockwool blocks saturated with nutrient solution and by the partial submergence of their root system, creating similar physiological consequences [21].

In order to shed light on the origin of the observed redox protective effect, we studied the expression of genes for antioxidant enzymes in control and treated plants. The expression of the selected genes is indicative of the ascorbate-glutathion cycle (APX and GR) and of the glutathione peroxidase cycle (GPX and GR). Despite expectations, we found a coordinated downregulation of gene coding for key components of antioxidant cycles upon Si treatment. This was in contrast with results described earlier in salt-stressed cucumber [9], where it was presumed that the observed changes in redox homeostasis may be caused by other metabolic alterations. We went on to investigate the potential involvement of pro-oxidant systems’ regulation in the observed effects of Si on the redox status. Amine oxidases (polyamine oxidases (PAOs), diamine oxidases (DAOs)) and lipoxygenases (LOXs) are well-known for their production of H_2_O_2_ and peroxygenated lipid species, respectively [13]. Association between polyamines and the role of Si in salt stress protection has been proposed [10] with the silicon-mediated downregulation of PAO or DAO activities. During our studies, however, a profound inhibitory effect of Si was also found on pro-oxidant PAO and DAO enzyme activities as well as on PAO gene expression in the cultivar hybrid ‘Joker’ subjected to a different type of stress. In silicon-treated plants, the total PAO and DAO activities declined, and so did the transcript level of several *PAO* and *LOX* genes. We could not identify candidate *DAO* genes in the genome of cucumber, although the corresponding enzyme activity was clearly present. A coordinated downregulation of *LOX* genes was also found in another cucumber hybrid in response to Si treatment, under different growth conditions [22]. This confirms this regulatory step as a likely common feature of Si action in cucumber. Association of Si with the cell wall and its effects on apoplast-related processes have been put forward [6,23]. Based on this association, extracellular space is an obvious compartment to look for potential mediators of silicate effects on redox balance. Polyamine and diamine oxidases are among known sources of apoplastic ROS [11]. 

According to the results presented here, Si treatment downregulated PAO and DAO activities and decreased H_2_O_2_ content with no associated growth promotion of the seedlings (Figure 1B). Similar to our results, silicon treatment also downregulated PAO activity in K-deficient sorghum, resulting in decreased putrescin and H_2_O_2_ levels and the alleviation of stress symptoms [24]. Mutations of *PAO* genes in the model plant Arabidopsis also caused the reduction of ROS levels [25]. 

The presented results suggest a negative impact of Si on enzymatic systems producing reactive oxygen species. Whether or not the downregulation of these enzyme activities affects transcription is not yet clear and deserves further attention. One possible scenario is that Si acts indirectly on gene expression by interfering with metabolism and/or the cellular transport of polyamines, which may well lead to profound and diverse effects on gene regulation [26]. Further on, the interference of Si with polyamine metabolism and transport may also be indirect. Si might modulate apoplastic processes associated with systemic stress responses similar to what was found in a case of a biotic interaction in tomato [27]. 

Apart from these uncertainties, our data definitely prove that silicon can modulate pro-oxidant enzymatic processes, uncovering the large regulatory potential of this effect. We have to note, however, that the actual effects of Si on diverse physiological and signaling processes are likely to be variable depending on the species, genotypes, and conditions/treatments. 

## 4. Materials and Methods

### 4.1. Plant Growth and Silicate Treatment

The open field-grown cucumber F1 cultivar hybrid ‘Joker’ was considered and used in this research. Seeds were obtained from Royal Sluis Magrovet (Hungary), submerged in 100 mL of distilled water for 24 h at 25 °C to imbibe. Four seeds were planted in 7.5 × 7.5 × 6.5 cm^3^ rockwool cubes and inserted into 20 cm diameter pots containing 200 g of perlite and grown at 26 ± 1 °C in a photoperiod of 16 h with a photosynthetic photon flux density of 150 µmol m^−^^2^ s^−1^ at culture level (provided by cool-white fluorescent lamps) and at 55–60% of relative humidity. The fertigation solution contained 2 mM potassium dihydrogen phosphate, 0.5 mM magnesium sulfate heptahydrate, 1.5 mM calcium sulfate dihydrate, 0.05 mM ferric sodium ethylenediaminetetraacetate, 0.5 mM morpholineethanesulfonic acid, 4 mM potassium nitrate, 0.572 mg.L^−1^ boric acid, 0.362 mg.L^−1^ manganese chloride tetrahydrate, 0.044 mg.L^−1^ zinc sulfate, 0.016 mg.L^−1^ copper sulfate, 0.004 mg.L^−1^ molybdenum trioxide. Silicate treatment was applied by fortification of the above solution with sodium silicate (1.67 mM). Control and Si supplemented fertigation (250 mL per pot) were applied every other day after expansion of cotyledons and the emergence of the first true leaves. Pots were partially (~2 cm) submerged in drained fertigation solution, with the pH adjusted daily to 6.0. Treatments continued for 21 days when fully expanded leaves were selected from each treatment. Growth rate was monitored by measuring the fresh weight of the shoot before processing leaf samples. Samples were taken to be used immediately or collected deep frozen in liquid nitrogen and stored at −80 °C for further molecular analysis. Two intact plants in each pot were analyzed with bio-photon emission imaging. All subsequent experiments were performed at least twice on different biological materials.

### 4.2. Ultra-Weak Bio-Photon Emission

Leaves of cucumber plants grown under Si treatments were subjected to ultra-weak photon emission imaging. Intact leaves of approximately the same size were detached from the plants and instantly placed in the chamber of NightShade LB 985 Plant Imaging System (Berthold Technologies, Bad Wildbad, Germany). Luminescence emissions from the leaves of control and Si-treated plants were imaged using thermoelectrically cooled (−70 °C) CCD camera (NightOWLcam, Berthold Technologies) mounted on a dark, light-tight chamber. A back-lit, midband-coated full frame chip with a spectral range of 350–1050 nm (quantum efficiency: 90% at 620 nm) was employed for photon detection and XY-imaging. The final resolution of 512 × 512 pixels and 26 × 26 µm^2^ pixel size (slow scan mode) were obtained by setting the variable binning to 2 × 2 and the exposure time to 60 s. IndiGo software (V. 2.0.5.0, Berthold Technologies, Germany) was used for image analysis. The presented images are selected from a series of photos taken and represent the highest detected signal intensity level in each treatment. 

### 4.3. Measurement of Si, H_2_O_2_, and Lipid Hydroperoxide Content, DAB Staining

Surface impurities were washed off the leaves with tap water after harvest. Si content was determined in leaf samples, which were dried at 37 °C for 2 days to obtain 5.0 g of the dried material from both Si-treated and control plants. Dry matter was ground to −200 mesh using a laboratory-type disc mill and 1.0 g of homogenized powder samples were incinerated in crucibles at 500 °C for 1 h to remove any organic matter. After cooling, 10 mL of 1 M HCl solution was added to the crucible, the mixture was filtered, and the crucible was washed twice with 10 mL of distilled water. The filtrate volume was then increased to 50 mL with distilled water. Optical emission spectroscopy of atoms excited by inductively coupled plasma (ICP-OES Ultima 2, HORIBA Jobin Yvon, Longjumeau, France) was employed at 251.921 nm and 252.851 nm wavelengths, results were expressed as mg.g^−1^ DW. H_2_O_2_, and lipid hydroperoxide content of the leaves were measured at 560 nm in the spectrophotometer, based on colorimetric reaction, as previously described by Kellős et al. [28]. Briefly, 200 mg of plant leaf samples were homogenized in 1 mL of 10% phosphoric acid. The supernatant was used for the determination of H_2_O_2_ and lipid hydroperoxides. 50 µL of the sample extract was mixed with a 950 µL mixture containing 100 µM Xylenol Orange, 250 µM ammonium ferrous sulphate, 100 µM sorbitol, and 25 µM sulfuric acid for H_2_O_2_ analysis or 100 µM Xylenol Orange, 250 µM ammonium ferrous sulphate, 90% methanol, 4 mM butylated hydroxytoluene, and 25 µM sulfuric acid for lipid hydroperoxide analysis. For both compounds H_2_O_2_ was used for calibration. In situ visualization of hydrogen peroxide level in leaves with DAB staining was carried out as described by Liu and Friesen [29]. Intact leaves were vacuum infiltrated for 10 min in a solution containing 1 mg.mL^−1^ 3,3-diaminobenzidine (DAB), 0.05% Tween 20, and 10 mM Na_2_HPO_4_ dissolved in water, the pH of which was previously adjusted to 3.6 with 0.2 M HCl. After 18 h of incubation leaves were bleached in ethanol/acetic acid/glycerol (3/1/1, *v*/*v*/*v*) solution in a boiling water bath for 10 min before imaging.

### 4.4. Amine Oxidases and Ascorbate Peroxidase Enzyme Activities

The activity of diamine oxidase (DAO, EC1.4.3.6.) and polyamine oxidase (PAO, EC 1.5.3.3.) enzymes was estimated by the method of Takács et al. [30], with minor modifications. A total of 300 mg of excised leaf tissues were homogenized in 0.9 mL of ice-cold extraction buffer containing 0.2 M Tris-HCl (pH 8.0), 10% glycerol, 0.25% Triton X-100; 0.5 mM phenylmethanesulfonyl fluoride (PMSF). The homogenate was left on ice for 20 min, then centrifuged (10 min, 12,000 rpm, 4 °C) and the supernatant was decanted. The reaction mixture (total volume of 830 µL) contained 0.15 mL of crude enzyme extract, 0.6 mL of 100 mM sodium phosphate buffer (pH 6.6), 7.5 µL of 1 M Putrescine for DAO, and 7.5 µL of 1 M Spermidine for PAO measurements. The reaction was carried out at 37 °C for 1.5 h and was stopped with 50 µL of 20% trichloroacetic acid (TCA). Subsequently, 22.5 μL of 10 mg.mL^−1^ 2-aminobenzaldehyde was added to the mixture, incubated on ice for 30 min, and centrifuged at 10,000× *g* for 10 min at 4 °C. Enzyme activity was measured at 430 nm in the spectrophotometer and expressed in nmol Δ1-pyrroline min^−1^.g^−1^ FW using an extinction coefficient of 1.86 × 103 mol−1.cm−1. Ascorbate peroxidase (APX, EC 1.11.1.1) activity was assayed according to Chandrakar et al. [31] by monitoring the rate of ascorbate oxidation at 290 nm. Leaf samples (200 mg) were homogenized in a 600 µL of extraction buffer (100 mM potassium phosphate buffer, pH 7.0, and 0.1 mM EDTA). The homogenate was centrifuged at 13,000 rpm for 15 min. One mL reaction mixture comprised of 50 mM phosphate buffer (pH 6.0), 0.1 µM EDTA, 0.5 mM ascorbate, 1.0 mM H_2_O_2_, and 50 µL enzyme extract. Enzyme activity was calculated following the extinction coefficient of 0.0028 M^−1^.cm^−1^ and expressed as µkat.g^−1^ FW.

### 4.5. RNA Isolation, cDNA Preparation, RT-PCR, and RT-qPCR

Total RNA was extracted from deep frozen leaf samples (collected from four leaves of the same size and position, from four different plants of different pots) of control and Si-treated plants by grinding in liquid N_2_ using sterile mortar and pestles. A modified CTAB-based protocol [32] was used to obtain high quality RNA as visualized on an EcoSafe-stained 1% agarose gel. RNA concentration was measured in a NanoDrop 1000 spectrophotometer and equalized to 10 μg per 100 μL in DNase I (Thermo Scientific) reaction mix. DNase-treated RNA integrity was ensured on agarose gel before reverse transcription. Five μg of total RNA was used for first-strand cDNA synthesis using the Maxima Reverse Transcriptase kit (Thermo Scientific) with oligo(dT)20 primers, according to the manufacturer’s protocol. Primers of selected redox-related genes and a control actin gene (Table S1.) were tested for PCR amplification with Dream Taq DNA polymerase (Thermo Scientific). Amplification was achieved in a Master Cycler instrument (Eppendorf AG, Hamburg, Germany) with 3 min at 95 °C and 26 cycles of 30 s: 95 °C, 60 s: 58 °C, 30 s: 72 °C, and a final extension for 7 min at 72 °C. Amplified genomic DNA and cDNA fragments were visualized on ethidium bromide-stained agarose gels. RT-qPCR was performed in a CFX 96 Real-Time PCR System (Bio-Rad, USA) using the SsoAdvanced Universal Inhibitor-Tolerant SYBR^®^ Green Supermix (Bio-Rad) for fluorescence detection in a 96-well optical plate. The total volume of each qPCR reaction was 10 μL, containing 1 μL of cDNA, 4 μL of super mix, 0.5 μL (100 uM) of forward and reverse primers, and 4 μL of PCR-grade water. Amplification was initiated with polymerase activation and DNA denaturation at 95 °C for 30 s, followed by 40 cycles of denaturation at 95 °C for 10 s, annealing and extension at 60 °C for 30 s. A melting curve analysis (65–95 °C) at the end of the run confirmed the specificity of the PCR products. Efficiency of PCR and stability of the endogenous standard gene (Actin-3 gene; DQ115883) were evaluated and sufficiently confirmed as described in Oszlányi et al. [33]. Fold changes of the examined gene expression were estimated by the 2^−∆∆Ct^ method [34].

### 4.6. Statistical Analysis

Data obtained from measurements were statistically analyzed with the statistical software IBM SPSS (v25) (IBM Corp, Armonk, NY, USA). Results are shown as mean values with standard deviations among three biological replicates. The normal distribution of residuals was proved by Shapiro–Wilk’s test (*p* > 0.05) and the homogeneity of variances was checked by Levene’s test (*p* > *α*) (*α* = 0.05). The differences among the mean values were evaluated by one-way ANOVA and considered significant at *p* < 0.05. The presented diagrams were produced in MS Excel.

## Supplementary Materials

The following are available online at https://www.mdpi.com/article/10.3390/plants10061248/s1, Table S1: Target genes, oligonucleotide primers, and expected product sizes in RT-PCRs and RT-qPCRs.
