# Supplementary material for: Downregulation of Polyamine and Diamine Oxidases in Silicon-Treated Cucumber
Source: Plants (Basel). 2021 Jun 19;10(6):1248. doi: 10.3390/plants10061248 (PMC8235019; doi:10.3390/plants10061248)
Supplement: Supplementary file 1 [file plants-10-01248-s001.zip › plants-1239745-supplementary.pdf]

Supplementary Materials

Downregulation of Polyamine and Diamine Oxidases in Silicon-Treated Cucumber

Anita Szegő[a§], Iman Mirmazloum[a§], Zsolt Pónya[abc], Oyuntogtokh Bat-Erdene[a], Mohammad Omran[a], Erzsébet Kiss-Bába[a], Márta Gyöngyik[a], István Papp[a]

Table S1. Target genes, oligonucleotide primers and expected product sizes in RT-PCRs and RT-qPCRs.

Gene	Accession No	Forward primer (5′-3′)	Reverse primer (5′-3′)	Amplicon size (bp)
CsaPAO1	CsaV3_7G030830	CCGTACTTGCTTCTCTGATTAC	GGGAGGATGCGGATGATTTA	157
CsaPAO2	CsaV3_4G032140	GGGATTACCTCTGTACCGTA	GCTAGTGACAAAAGTTGGGC	125
CsaPAO3	CsaV3_2G001470	GGAAGCCATTTTCGCCATGT	GCGTAATCGAATCAATCGCC	160
CsaPAO4	CsaV3_4G007490	CCGTCTGTTATAGTGATCGG	TGTGAGGAAAATCCGTTGGC	197
CsaAPX1	CsaV3_6G021870	CTTCGTCTCGCATGGCA	TGCCATTGAAGTTACTGGTGG	221
CsaAPX2	CsaV3_2G032090	CGACACGAGAACTAGTTGCACTATC	GAATGCTTGAGATGGATCAAAGAG	111
CsaGPX1	CsaV3_4G036450	ATCAAGTGCTGGAGGGTTT	GAGATACCCACCAACAACATC	105
CsaGR1	CsaV3_3G011090	TACGATCTCTGGCCGACAAGAG	CAGCACTGTTGGAATACACCCAT	182
CsaGR2	CsaV3_7G027540	CGTGGAAAGATTCTGGATGAAC	GCTCTAGAATTTGCTGGCATC	216
CsaLox1	CsaV3_2G006440	AGGACTTCCTGTTCCATTGGA	TCGTCTTAAGGAATTTCCTCCA	229
CsaLox4	CsaV3_2G006460	GCAACTCATGGGTTTACCC	GGAAAAGGAGAGCGTCAAG	156
CsaLox8	CsaV3_2G005360	TCCTGCTGACAAATACAAAACC	CTTTTTGGATCTTTTTTGGAAGG	297
CsaLox10	CsaV3_2G005350	TGGGAGAAGAAGCATACTTAGAGG	GGAAGATAGCTCTGGTTGAGAAAG	286
CsaLox17	CsaV3_7G027160	CCTCGTCGAATAAGAACTCTTC	CCCTAAACTATCAACCTTCCAC	180
CsaLox19	CsaV3_4G023920	CTCATTGGCTGAGAACTCATTG	TGAATTAAGTCAGCAGGTAAAGCT	271
CsaLox20	CsaV3_4G023930	TTGCTTCCAATAAGATGGGAC	TCCCGTTACAGGATCAACCT	264
CsaLox22	CsaV3_7G034390	TCTCTAATAAGCCTTATCTTCCAGGT	TGCAGTCATATCAGTCTCGGA	260
CsaLox23	CsaV3_5G030460	TAAATCATTGGTTAAGGACTCACG	CCATACCCCTTCGAATAAGATC	285
Actin	CsaV3_2G018090	TCGTGCTTGACTCTGGTGATGG	ACAACCACTGCCGAACGGGAAA	171
